# Infant Food Responsiveness in the Context of Temperament and Mothers' Use of Food to Soothe

**DOI:** 10.3389/fnut.2021.781861

**Published:** 2022-01-11

**Authors:** Holly A. Harris, Amy M. Moore, Cara F. Ruggiero, Lisa Bailey-Davis, Jennifer S. Savage

**Affiliations:** ^1^Generation R Study Group, Erasmus MC, University Medical Center, Department of Child and Adolescent Psychiatry/Psychology, Rotterdam, Netherlands; ^2^Department of Human Health and Development, Center for Childhood Obesity Research, The Pennsylvania State University, University Park, PA, United States; ^3^Population Health Sciences, Obesity Institute Geisinger, Danville, PA, United States

**Keywords:** appetite, emotional feeding, food to soothe, food responsiveness, infant feeding, low income, self-regulation, temperament

## Abstract

Parents' use of food to soothe an infants' non-hunger related distress may impair an infants' development of appetite self-regulation. Parents tend to use food to soothe if their infant has more ‘difficult' temperamental tendencies. However, the role of infant appetite in this association is unclear. This study investigates the moderating effect of infant food responsiveness on cross-sectional and prospective associations between infant temperament and mothers' use of food to soothe. Mothers (*n* = 200) from low-income households reported their infants' temperament (i.e., surgency, negative affect and regulation) and food responsiveness at age 4 months, and their use of food to soothe at age 4 and 6 months. Temperament × food responsiveness interactions on mothers' use of food to soothe were examined using general linear models, adjusting for covariates. Cross-sectional associations showed that mothers used more food to soothe at 4 months for infants who were lower in negative affect and higher in food responsiveness (negative affect × food responsiveness interaction: *p* = 0.03). Prospective associations showed that mothers used more food to soothe at 6 months for infants who were lower in regulation and higher in food responsiveness (infant regulation × food responsiveness interaction: *p* = 0.009). Other interactions were not significant. Infant food responsiveness was consistently associated with mothers' use of food to soothe, independent of some temperamental dimensions. The findings highlight the salience of infant food responsiveness, both independent of and in association with temperament, on mothers' use of food to soothe.

## Introduction

Individual differences in appetite self-regulation (ASR) emerge through a complex interplay of biological predispositions, the psychosocial environment, and wider sociocultural factors (1). ASR broadly describes feedback mechanisms that ‘cue' individuals to eat to meet their nutritional and energy needs (2). One mechanism includes internal signaling hunger and fullness cues to encourage individuals to start and stop eating, respectively. An individual's phenotypic appetitive tendencies, or ‘appetitive traits', are intrinsically linked to ASR (3). One such trait is ‘food responsiveness', which in developmental terms may represent a bottom-up, approach-related response to food and food cues (3). Food responsiveness describes an individuals' affinity to food and eating, responsiveness to external food cues, such as the sight, smell and taste of palatable foods (4). Individual differences in food responsiveness are apparent from infancy, and increased food responsiveness has been associated with rapid weight gain and overweight (5–7). While appetitive traits are genetically influenced, psychosocial environmental factors, such as parenting and the child-parent relationship, also interact with the expression of these phenotypes (8). Moreover, epidemiological evidence suggests that socioeconomic disadvantage predicts increased expression of food responsiveness from toddlerhood to the preschool period (9). While the origins of ASR remains unclear, research rarely considers the *context* in which it is expressed and evolves. Contextual understanding of the origins of ASR requires a thorough understanding of individuals' predispositions, and how this interacts with their immediate environment, beginning in infancy (10).

Young infants rely on their caregivers (most often their parents) to regulate their physical and emotional needs. This includes responding to their infants' hunger and fullness cues and providing opportunities for them to practice and develop self-regulatory skills. Just like the dynamic infant-parent interactions that contribute to an infants' social and emotional development (11), so too are feeding interactions likely to impact an infants' ASR development. Black and Aboud (12) suggest that parents' responsive feeding practices align with infant hunger and fullness cues to support infant development of eating autonomy. Conversely, controlling feeding practices may (unintentionally) override a child's ability to start and stop eating in response to internal signals of hunger and fullness, and thus disrupt ASR (12). An example of a controlling feeding practice **–** and the focus of this current study **–** is parents' use of food to soothe a non-hungry but distressed infant. While effective in the short-term, parents' use of food to soothe may have unintended consequences on the development of ASR. For example, infants may learn that negative emotions are attenuated with the pleasurable effects of feeding, while parents may learn that feeding has a potent, calming effect on an infants' emotional state (13). This may minimize an infant's opportunities to build self-soothing skills (14) which is component of general self-regulation. Furthermore, parents' use of food to soothe has been associated with children's emotional overeating (15) and weight gain (16, 17), which may indicate a disruption of ASR. Use of food to soothe is observed more frequently in mothers from low-income households or with lower levels of education (18, 19). Therefore, examining this feeding interaction in populations where use is intensified may illuminate processes involved in ASR development.

Feeding interactions involve complex and bidirectional transactional processes between infant and parent (20). In other words, parent feeding practices influence infants' eating or behaviors, and so too do infant characteristics influence parent feeding practices. Infant temperament, for example, may play a role in evoking parents' use of food to soothe (21, 22). Temperament refers to individual differences in behavior that are biologically based or inborn, and is characterized by an infants' reactivity and self-regulation (23). Broad dimensions of temperament include surgency (high activity and approach to novelty/reward), negative affect (fussiness/ crying) and orienting/ regulation (effortful control, ability to self-regulate emotions or focus attention) (24). These temperamental traits in infancy may be precursors to ‘top-down' regulatory processes involved in general self-regulation, and possibly, ASR (3). Cross-sectional studies demonstrate that infants or children who tend to express more ‘difficult' temperamental tendencies have parents who use feeding as a soothing mechanism (21, 22). Despite the established associations between temperament and parents' use of food to soothe, the role of appetite in this association remains unclear. Similarly to food responsiveness, aspects of child temperament have also been identified as a risk factor for weight gain and obesity (8).

Although temperament and appetitive traits are two distinct constructs (23), appetitive traits are hypothesized to manifest through temperament. This has been prospectively demonstrated from early to middle childhood (25). Scant evidence shows that mothers of infants higher in food responsiveness are more likely to report using food to soothe (26). This suggests that parents may learn that feeding is an effective soothing tool for infants who respond favorably to food. It is therefore conceivable that the association between difficult infant temperament and parents' use of food to soothe may be exacerbated for infants higher in food responsiveness. In other words, infant temperament and food responsiveness may interact in a ‘top down, bottom up' model which may contribute to parents' use of food to soothe and bidirectionally influence ASR.

The current study examines how infant characteristics (i.e., temperament and food responsiveness) are associated with mothers' use of food to soothe. We examine these associations in a sample of mother-infant dyads from low-income households. As early infancy marks a period of rapid development and dietary transitions, we examined the associations of infant temperament and food responsiveness at 4 months on mothers' (i) concurrent use of food to soothe at infant age 4 months, and (ii) prospective use of food to soothe at infant age 6 months, to examine how these effects may manifest over this period. The relationship between temperaments that are perceived to be more ‘difficult' (greater surgency and negative affect, lower orienting/regulation) and mothers' use of food to soothe was hypothesized to be stronger for infants with greater food responsiveness, both cross-sectionally and prospectively.

## Methods

### Study Design and Population

The current study is a secondary analysis of mother-infant dyads from the WEE Baby Care study, a pragmatic randomized clinical trial (RCT) designed to promote responsive parenting and to prevent rapid infant weight gain (27). Detailed information regarding the study design has been published (27). Briefly, mothers and their newborn infants were recruited from July 2016 to May 2018 in northeastern Pennsylvania, an area geographically characterized as Medically Underserved by the Health Services and Resources Administration (28). Mother-infant dyads were recruited if they attended Special Supplemental Nutrition Program for Women, Infants and Children (WIC) clinics and well-child visits (WCVs) at pediatric Primary Care Providers (PCPs) that participated in the study. Mother-infant dyads were excluded if: there were plans for the newborn to be adopted, the newborn's birth weight was <2500 g, the mother anticipated switching to a non-participating provider within 6–9 months, they did not live in the service area of the participating WIC clinics, or either mother or infant had significant health issues that would affect study participation or feeding and/or growth. Enrolled dyads were randomized into a 6-month responsive parenting intervention group (*n* = 131) or a standard care control group (*n* = 157). In addition, care for mother-infant dyads assigned to the responsive parenting intervention group was coordinated and integrated across pediatric PCPs and WIC settings using advanced Health Information Technology strategies (29). This study was approved by the Institutional Review Boards of The Pennsylvania State University and Geisinger. All participants provided written informed consent.

Mothers completed surveys at three time points when infants were approximately aged 2, 4 and 6months. The current study examines dyads with data on infant temperament and food responsiveness at infant age 4 months, and mothers' use of food to soothe at infant age 4 (*n* = 199) and 6 months (*n* = 200; one participant had missing data on food to soothe at 4 months). Mothers who were excluded due to missing data on the variables of interest (*n* = 88) were younger and came from lower income households compared to the analytic sample (*n* = 200). Infant birth date and date of assessment completion were used to calculate infant age in months at each time point.

### Measures

#### Sociodemographic Characteristics

Participants completed surveys online through the REDCap electronic survey system (30) or paper questionnaires. Demographic variables were collected from mothers at enrollment, including age, marital status, highest level of education attained, employment status, household income and number of people living in the household. Infant sex, gestational age and birth weight were obtained from patient electronic health records (EHR). Infant anthropometric data was assessed by trained staff in pediatric PCP clinics at infant age 6 months. Mothers reported their infant's race and ethnicity. At infant age 4 and 6 months, mothers reported whether they were exclusively breast feeding and whether they had introduced solid foods (i.e., complementary feeding).

#### Infant Temperament

At infant age 4 months, mothers reported on their infant's temperament using the Infant Behavior Questionnaire (IBQ - Revised) – Very Short Form (31). The current study examines three broad temperament dimensions on the IBQ: positive affectivity/surgency (herein referred to as ‘surgency'), ‘negative affect' and ‘orienting/ regulation' (herein referred to as ‘regulation'). Surgency relates to an infant's approach to novelty, activity level, vocal reactivity, high intensity pleasure, smiling/ laughter and perceptual sensitivity (13 items; α=0.76, e.g. ‘*During a peekaboo game, how often did [your] baby laugh?*'). Negative affect describes an infants' tendency to express fear, sadness, anger and discomfort (12 items; α = 0.80, e.g., ‘*When tired, how often did your baby show distress?'*). Regulation assesses an infants' soothability, cuddliness, attention abilities, inhibitory control and low-intensity pleasure (12 items; α = 0.77, e.g., ‘*When patting or gently rubbing some part of the baby's body, how often did s/he soothe immediately?'*). Mothers responded to items on 7-point scale from never (1) to always (7) and scores were averaged within each subscale. Higher mean scores indicated greater levels of that temperament dimension.

#### Infant Food Responsiveness

At infant age 4 months, mothers completed the food responsiveness subscale of the Baby Eating Behavior Questionnaire (BEBQ) (4). Food responsiveness is assessed with 5 items asking about an infant's responsiveness to cues of milk and feeding, and drive to feed (e.g. ‘*My baby was always demanding a feed'*). Mothers responded to items on a 5-point scale from never (1) to always (5). Items were averaged to produce a mean score, with higher scores indicating greater food responsiveness (α = 0.83).

#### Mothers' Use of Food to Soothe

At infant age 4 and 6 months, mothers self-reported their use of food to soothe infant distress using 12 items from a modified version of the Baby's Basic Needs Questionnaire (21, 32). Mothers responded to items (*e.g. ‘How likely are you to use food (breastmilk, formula, other drinks or foods) to calm your child when you are shopping in a store?'*) on a 5-point scale from never (1) to always (5). Items were averaged to create a mean score with higher scores indicating mothers' greater use of food to soothe (4 months α = 0.87; 6 months α = 0.85).

### Statistical Analysis

Data were analyzed using SAS 9.4 (SAS Institute, Cary, NC). Statistical significance was defined as *p* < 0.05, and all inferential tests were 2-sided. Sociodemographic characteristics and the main variables of interest were compared by study group using independent samples *t*-tests and χ^2^ tests for continuous and categorical variables, respectively. There were no differences by study group on any sociodemographic factors or other main variables of interest (Supplementary Table 1). Descriptive statistics on the main variables of interest were run and assessed for normality. Pearson correlations examined bivariate associations between main study variables. General linear models were run to examine the interaction between temperament (one model for each dimension: surgency, negative affect, and regulation) and food responsiveness on mothers' use of food to soothe. Due to the small sample size and strong correlation between food to soothe at 4 and 6 months (*r* = 0.78, *p* < 0.001), cross-sectional (4 months) and prospective associations (6 months) were examined separately. Models adjusted for maternal age, education and marital status, and exclusive breastfeeding and introduction to solids. Missing data on covariates (≤ 6% missing) were imputed using the Markvo chain Monte Carlo multiple imputation method. Analyses were based on pooled results of 10 imputed datasets. Statistically significant interactions were probed and plotted to facilitate the interpretation of results. If an interaction was not statistically significant, the main effects of the infant temperament dimension and food responsiveness on mothers' use of food to soothe were examined using general linear models. In a sensitivity analysis, all models were rerun adjusting for study group. However, this did not significantly alter the results. Therefore, models unadjusted for study group are reported.

## Results

Sociodemographic characteristics of mother-infant dyads are shown in Table 1. Mothers were mostly white, single or divorced and high school educated (or less). Bivariate correlations between the main variables of interest are shown in Supplementary Table 2. Results for the main analyses are shown in Table 2, and are discussed by each infant temperamental trait below.

**Table 1:** (Non-imputed) participant characteristics (*N* = 200).

	***n* (%) or mean ± SD**	***n* total data available**
**Infant**		
Male	99 (49.5)	200
Gestational age, weeks	39.2 (1.1)	200
Birth WFL *z* score	0.7 ± 1.3	198
WFL *z* score, age 6 months	0.5 ± 1.1	153
Exclusively breastfed, age 4 months	34 (17.5)	194
Exclusively breastfed, age 6 months	28 (14.6)	192
Introduced to solid foods, age 4 months	127 (65.5)	194
Introduced to solid foods, age 6 months	186 (95.9)	194
Temperament^a^, age 4 months (scale 1 to 7)		
Surgency	5.0 ± 0.9	200
Negative affect	3.2 ± 1.0	200
Regulation	5.7 ± 0.7	200
Food responsiveness^b^, age 4 months (scale 1 to 5)	1.8 ± 0.7	200
**Mother**		
Age at infant birth, years	28.1 ± 5.5	188
Marital status		189
Married and/or living with partner	92 (48.7)	
Single/Divorced	97 (51.3)	
Educational level		189
High school or less	117 (61.9)	
Some college	52 (27.5)	
College graduate or greater	20 (10.6)	
Annual household income		176
<$10,000	44 (25.0)	
$10,000–$24,999	68 (38.6)	
$25,000–$49,999	59 (33.5)	
$50,000–$74,999	5 (2.8)	
Race		200
Black	28 (13.5)	
White	137 (68.5)	
Other	36 (18.0)	
Hispanic	41 (21.8)	188
Average size of household, persons	3.2 ± 1.4	184
Food insecure	56 (29.0)	193
Food to soothe^c^, infant age 4 months (scale 1 to 5)	2.3 ± 0.7	199
Food to soothe^c^, infant age 6 months (scale 1 to 5)	2.2 ± 0.7	200

*WFL, Weight-for-Length*.

a *Infant temperament measured via the Infant Behavior Questionnaire-R Very Short Form (31)*.

b *Baby Eating Behavior Questionnaire (4)*.

c *Modified version of the Baby's Basic Needs Questionnaire (21)*.

**Table 2:** General linear models showing the associations between infant temperament dimension^a^ and food responsiveness^b^ at 4 months on mothers' use of food to soothe at 4 and 6 months^c^.

	**Temperament (4 months)**					
	**Surgency**		**Negative affect**		**Regulation**	
**Cross-sectional outcome:** **Mothers' use of food to soothe (4 months)**	**B (SE)**	***p***	**B (SE)**	***p***	**B (SE)**	***p***
Temperament	0.09 (0.05)	0.09	0.34 (0.12)	0.006	0.06 (0.06)	0.37
Food responsiveness	0.43 (0.07)	<0.0001	0.85 (0.23)	0.0002	0.46 (0.07)	<0.0001
Temperament × Food responsiveness	-	-	−0.13 (0.06)	0.03	-	-
Model R^2^	0.24		0.26		0.23	
F statistic	7.54		7.53		7.22	
*p* value	<0.0001		<0.0001		<0.0001	
**Prospective outcome:** **Mothers' use of food to soothe (6 months)**	**B (SE)**	***p***	**B (SE)**	***p***	**B (SE)**	***p***
Temperament	0.10 (0.05)	0.07	0.14 (0.05)	0.007	0.43 (0.19)	0.023
Food responsiveness	0.35 (0.07)	<0.0001	0.34 (0.08)	<0.0001	1.75 (0.54)	0.001
Temperament × Food responsiveness	-	-	-	-	−0.24 (0.10)	0.013
Model R^2^	0.21		0.22		0.22	
F statistic	6.28		6.92		5.98	
*p* value	<0.0001		<0.0001		<0.0001	

*Cross-sectional models adjust for maternal age, education, marital status, and exclusive breastfeeding and introduction to solids at infant age 4 months; Prospective models adjust for maternal age, education, marital status, and exclusive breastfeeding and introduction to solids at infant age 6 months*.

a *Infant temperament measured via the Infant Behavior Questionnaire-R Very Short Form (31)*.

b *Baby Eating Behavior Questionnaire (4)*.

c *Baby's Basic Needs Questionnaire (21)*.

### Surgency and Food Responsiveness

For both cross-sectional and prospective associations with mothers' use of food to soothe, the infant surgency × food responsiveness interaction was not statistically significant. In the main effects model, food responsiveness at 4 months was positively associated with mothers' use of food to soothe at both 4 months and 6 months, independent of surgency at 4 months, which was not statistically significant in both models.

### Negative Affect and Food Responsiveness

**Figure 1:**
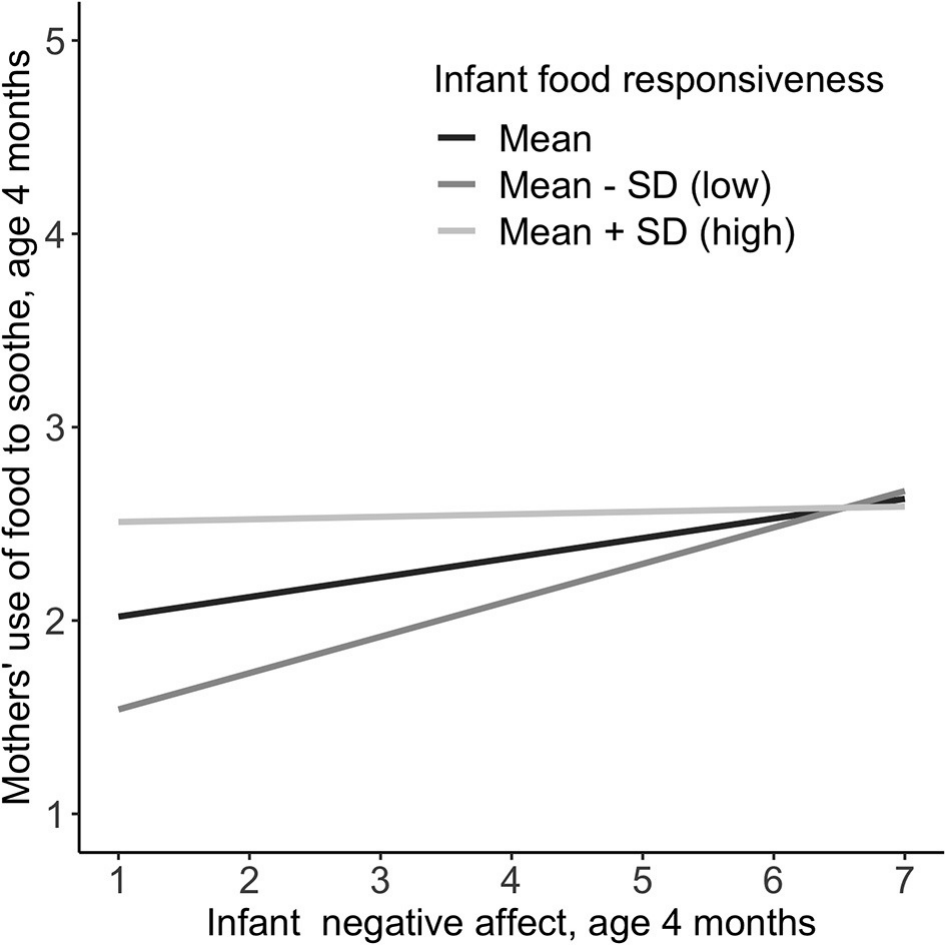
Interaction between infant negative affect and relative levels of infant food responsiveness at 4 months on mothers' cross-sectional use of food to soothe at c months. Negative affect measured *via* the Infant Behavior Questionnaire-R Very Short Form (31); Food responsiveness measured *via* the Baby Eating Behavior Questionnaire (4). Mean = 1.8, SD = 0.7 (scale 1–5); Food to soothe measured *via* the Baby's Basic Needs Questionnaire (21).

Cross-sectionally, the infant negative affect × food responsiveness interaction on mothers' use of food to soothe was statistically significant. Simple slope analysis indicated that the slope of infant negative affect on mothers' use of food to soothe depends on infant food responsiveness. Figure 1 presents the effect of negative affect on maternal food to soothe at 3 levels of infant food responsiveness based on the mean, mean – SD (low) and mean + SD (high). The figure shows that infants lower in negative affect and higher in food responsiveness have mothers who use food to soothe more frequently. Prospectively, the infant negative affect × food responsiveness interaction on mothers' use of food to soothe at 6 months was not statistically significant. However, the main effects model showed that both infant negative affect and food responsiveness were independently and positively associated with mothers' use of food to soothe.

### Regulation and Food Responsiveness

**Figure 2:**
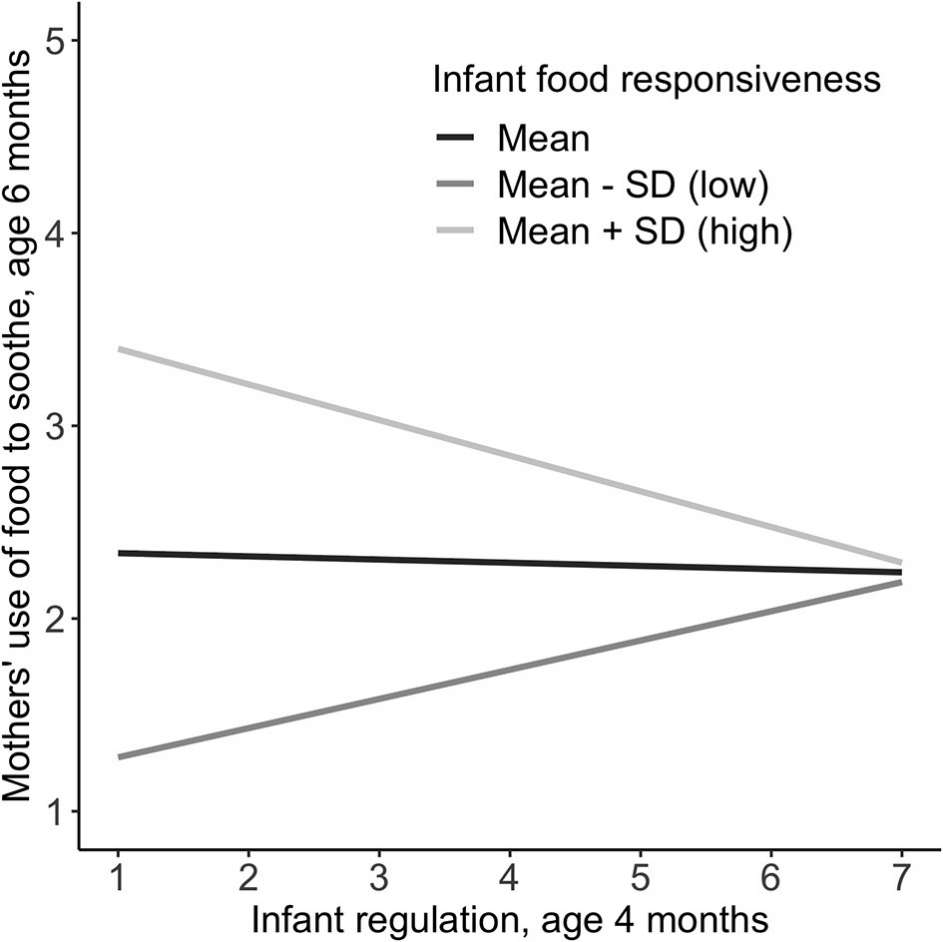
Interaction between infant regulation and relative levels of infant food responsiveness at 4 months on mothers' prospective use of food to soothe at 6 months. Regulation measured *via* the Infant Behavior Questionnaire-R Very Short Form (31); Food responsiveness measured *via* the Baby Eating Behavior Questionnaire (4). Mean = 1.8, SD = 0.7 (scale 1–5); Food to soothe measured *via* the Baby's Basic Needs Questionnaire (21).

Cross-sectionally, the interaction between infant regulation × food responsiveness was not associated with mothers' use of food to soothe. The cross-sectional main effects model showed that food responsiveness was positively associated with mothers' use of food to soothe, independent of regulation, which was not statistically significant. Prospectively, the interaction between infant regulation × food responsiveness on mothers' use of food to soothe was statically significant, and therefore simple slopes were examined. Figure 2 presents the effect of infant regulation on mothers' use of food to soothe at three levels of infant food responsiveness. The figure shows that children low in regulation and high in food responsiveness have mothers who use food to soothe more frequently at 6 months.

## Discussion

The current study expands the field's understanding of processes involved in the developmental origins of ASR. Our findings generally replicate previous research in middle-income families that demonstrate a positive association between infant ‘difficult' temperament and mothers' use of food to soothe (21, 22). We also extend these findings by demonstrating the salient role of infant food responsiveness in this association, in a sample from low-income households. Findings highlight the independent and interrelated role of infant temperament and food responsiveness on mothers' use of food to soothe, which could ultimately shape children's development of ASR. We found that mothers respond dynamically to multiple facets of their infant's characteristics when using food to soothe infant distress. Specifically, at infant age 4 months, food responsiveness moderated the association between negative affect and mothers' concurrent use of food to soothe. Similarly, we also showed a moderating effect of infant food responsiveness on the association between regulation at age 4 months and mothers' use of food to soothe at 6 months. In addition, food responsiveness was consistently independently associated with mother's use of food to soothe. This also replicates findings in older children showing a link between food responsiveness and food to soothe (33). Future responsive parenting interventions could consider identifying ‘at-risk' participants who are high on food responsiveness and difficult temperamental traits, and develop unique messaging based on these traits.

Stifter and colleagues instigated research on child temperament and mothers' use of food to soothe, and associations on child weight. Their early cross-sectional work showed that child negative affect was positively associated weight, and this association was intensified by mothers' increasing use of food to soothe (21). In the current study, the effect of infant negative affect on mothers' concurrent use of food to soothe was intensified with increasing food responsiveness. Interestingly, both negative affect and food responsiveness were independently associated with mothers' use of food to soothe at age 6 months. While the correlational nature of our analyses precludes interpretations about directionality, it is possible that the effects of temperament and food responsiveness on mothers' food to soothe evolve over time. A nuanced understanding of how infant negative affect and food responsiveness are associated with mothers' evolving use of food to soothe in the first 6 months of life is needed.

Despite no association between infant regulation and mothers' concurrent use of food to soothe, the association between regulation and mothers' prospective use of food to soothe was dependent on food responsiveness. This is also suggestive of the evolving role of mothers' responding to infant characteristics over time. While many studies have demonstrated associations between negative affect or surgency and food to soothe (21, 34), a renewed focus on regulation may be equally important at a very young age. Regulation may be precursor to later emerging effortful control (35), which is a part of general self-regulation related to top-down self-regulatory processes (3, 36). Current findings show that infants who may be lower in soothability and duration of orienting but who also respond favorably to external food cues, appear to have mothers who use feeding to calm their infants. Self-soothing abilities rapidly increase across the first year of life (37). Therefore, feeding to regulate an infants' emotional state during this period of developmental plasticity could promote maladaptive eating behaviors that contribute to appetite dysregulation later in childhood, such as emotional overeating (15). Interventions could focus on supporting mothers in identifying behaviors which indicate low orienting or regulatory capacity (e.g., difficulty in soothing or sustaining attention on an object) and high food responsiveness (e.g., frequently demanding feeds, taking feeds when offered or always preferring to be fed). For infants with these tendencies, mothers may require additional support to engage in alternative soothing strategies (38) rather than feeding.

Stifter's more recent work showed that infant surgency was prospectively associated with increased weight gain in toddlerhood when mothers used more food to soothe (34). However, we found that infant surgency was not associated with mothers' use of food to soothe cross-sectionally or prospectively when accounting for infant food responsiveness. Food responsiveness was independently associated with mothers' use of food to soothe at both 4 and 6 months. In contrast to negative affect, surgency is generally characterized by high positive affect, activity level and extraversion, which may not necessarily ‘evoke' mothers' use of food to soothe when accounting for food responsiveness in infancy. It is also possible that these dynamics evolve throughout child development, with potentially adverse consequences on ASR and future weight gain (34). Therefore, further longitudinal research is required to examine how these predispositions change over time and are associated with mothers' use of food to soothe.

This research advances the understanding of one of many complex and interconnected pathways involved in the development of ASR. Based on our results, we propose two possible – and testable – mechanisms at play in the development of ASR. Firstly, our research is suggestive of an evolving and transactional infant-parent feeding processes which can potentially impinge on the infants' experiences in responding to their appetite and environmental food cues, and thus, ASR. The transactional model of development proposes that parents and infants engage in bi-directional interactions (39). In feeding, the infant plays an active role in shaping transactions through their hunger and fullness cues (i.e., appetite), which may indicate a physiological need filtered through their temperamental disposition (40). Simultaneously, the parent actively responds to the infant's cues, initiating and terminating feeding which then shapes the infant's intake and subsequent ASR. Secondly, we propose that a combination of inherent temperamental and appetitive traits (for example: regulation, negative affect and food responsiveness), may co-act as ‘susceptibility factors' which are related to parents' use of food to soothe. Belsky's model of differential susceptibility (41) proposes that children with certain characteristics may be more susceptible to adverse or beneficial environmental impacts (e.g. parenting) which, in turn, influences an outcome. Applied to findings in the current study, it is possible that greater food responsiveness and more ‘difficult' temperamental traits interact and render an individual more susceptible to parents' use of food to soothe, and may impede a child's developing ASR. This highlights the need for future research to examine the origins of ASR within the parent feeding context. Future research can extend this work by examining parent's feeding practices and styles, as well as their cognitions, interpretations and expectations in the realm of child eating behaviors (42).

Strengths of the current study must be considered in light of certain limitations. While much of the previous research has focused on middle income families, we examined a community sample of mothers and their infants from low-income households, who may be at greater risk of obesity. However, mothers were mostly white and additional work in needed in more racially diverse samples. This study is also a secondary data analysis of mothers recruited in a clinical trial and therefore introduces selection bias. Shared variance bias may be introduced through the use of maternal self-report of some variables. However, constructs used were derived from validated and widely-used questionnaires. For example, the BEBQ has been validated against objective measures of eating behavior (43, 44) and mothers are in a good position to report on their child's appetite over time, as opposed to a one-time observational measure (45). Due to the correlational nature of our measures and analysis, our results do not imply directionality. Future research could consider teasing apart mechanisms underlying the relationship between infant temperament, food responsiveness and mothers' use of food to soothe, longitudinally across infancy and early childhood.

Results from the current analysis reveal that both infant food responsiveness and temperament dynamically contribute to mothers' use of food to soothe. Although conceptually distinct, temperament and appetitive traits are closely intertwined factors associated with infant feeding interactions with parents. Teaching non-food soothing strategies to mothers and developing evidence-based messaging tailored to the unique characteristics of the infant could be a potential direction for future intervention. Identifying children with “high-risk” temperaments and appetitive traits may allow for opportunities to teach mothers responsive feeding strategies to prevent child weight gain across clinical and community settings in low-income populations.

## Data Availability Statement

The original contributions presented in the study are included in the article/Supplementary Materials, further inquiries can be directed to the corresponding author.

## Ethics Statement

The studies involving human participants were reviewed and approved by Institutional Review Boards of The Pennsylvania State University and Geisinger. The patients/participants provided their written informed consent to participate in this study.

## Author Contributions

HAH conceptualized the study, designed the analysis, and drafted the original manuscript. AMM conducted the analysis. HAH, AMM, CFR, and JSS interpreted the results. AMM and CFR contributed to editing the manuscript. LB-D and JSS edited the manuscript, provided important intellectual content, and were involved in the funding acquisition. All authors have read and agree to take responsibility for the content of the manuscript.

## Funding

This project is supported by the Health Resources and Services Administration (HRSA) of the U.S. Department of Health and Human Services (HHS) under grant number R40MC28317, Maternal and Child Health Field-initiated Innovative Research Studies Program. Funding for REDCap was provided by The Penn State Clinical & Translational Research Institute, Pennsylvania State University CTSA, NIH/NCATS Grant Number UL1 TR002014).

## Conflict of Interest

The authors declare that the research was conducted in the absence of any commercial or financial relationships that could be construed as a potential conflict of interest.

## Publisher's Note

All claims expressed in this article are solely those of the authors and do not necessarily represent those of their affiliated organizations, or those of the publisher, the editors and the reviewers. Any product that may be evaluated in this article, or claim that may be made by its manufacturer, is not guaranteed or endorsed by the publisher.
