# Supplementary material for: Infant Food Responsiveness in the Context of Temperament and Mothers' Use of Food to Soothe
Source: Front Nutr. 2022 Jan 11;8:781861. doi: 10.3389/fnut.2021.781861 (PMC8786708; doi:10.3389/fnut.2021.781861)
Supplement: Supplementary file 1 [file Table_1.docx]

**Supplementary Material**

**Table S1.** Participant characteristics by study group

| **Infant** | *n* (%) or mean ± SD | | *p* value |
| --- | --- | --- | --- |
|  | Intervention  (*n*=93) | Control  (*n*=107) |  |
| Male | 47 (50.5) | 52 (48.6) | 0.78 |
| Gestational age, weeks | 39.2 ± 1.1 | 39.2 ± 1.2 | 0.79 |
| Birth WFL *z* score | 0.8 ± 1.3 | 0.6 ± 1.3 | 0.33 |
| Exclusively breastfed, age 4 months | 20 (10.0) | 14 (7.0) | 0.11 |
| Exclusively breastfed, age 6 months | 16 (8.0) | 12 (6.0) | 0.22 |
| Introduced to solid foods, age 4 months | 31 (33.3) | 36 (33.6) | 0.97 |
| Introduced to solid foods, age 6 months | 90 (96.8) | 102 (95.3) | 0.60 |
| Temperament^a^, age 4 months (scale 1 to 7) |  |  |  |
| Surgency | 4.9 ± 0.9 | 5.1 ± 0.9 | 0.48 |
| Negative affect | 3.2 ± 0.9 | 3.3 ± 1.1 | 0.06 |
| Regulation | 5.7 ± 0.8 | 5.7 ± 0.7 | 0.88 |
| Food responsiveness^b^, age 4 months (scale 1 to 5) | 1.8 ± 0.7 | 1.9 ± 0.7 | 0.84 |
| WFL *z* score, age 6 months | 0.6 ± 1.2 | 0.5 ± 1.0 | 0.53 |
| **Mother** | | | |
| Age at infant birth, years | 28.3 ± 5.7 | 27.9 ± 5.3 | 0.53 |
| Marital status |  |  |  |
| Married and/or living with partner | 40 (44.9) | 52 (52.0) | 0.33 |
| Single/Divorced | 49 (55.1) | 48 (48.0) |  |
| Educational level |  |  | 0.97 |
| High school or less | 55 (61.8) | 62 (62.0) |  |
| Some college | 25 (29.1) | 27 (27.0) |  |
| College graduate or greater | 9 (10.1) | 11 (11.0) |  |
| Annual household income |  |  | 0.55 |
| < $10,000 | 22 (26.2) | 22 (23.9) |  |
| $10,000-$24,999 | 36 (42.9) | 32 (34.8) |  |
| $25,000-$49,999 | 24 (28.6) | 35 (38.0) |  |
| $50,000-$74,999 | 2 (2.4) | 3 (3.3) |  |
| Race |  |  | 0.07 |
| Black | 17 (18.3) | 10 (9.4) |  |
| White | 64 (68.8) | 73 (68.2) |  |
| Other | 12 (12.9) | 24 (22.4) |  |
| Hispanic | 19 (10.1) | 22 (11.7) | 0.94 |
| Average size of household, persons | 3.3 ± 1.6 | 3.0 ± 1.3 | 0.24 |
| Food insecure, yes | 28 (31.1) | 28 (27.2) | 0.55 |
| Food to soothe^c^, infant age 4 months (scale 1 to 5) | 2.2 ± 0.7 | 2.3 ± 0.7 | 0.81 |
| Food to soothe^c^, infant age 6 months (scale 1 to 5) | 2.1 ± 0.7 | 2.2 ± 0.8 | 0.66 |

WFL: Weight-for-Length; ^a^Infant temperament measured via the Infant Behavior Questionnaire-R Very Short Form (31); ^b^Baby Eating Behavior Questionnaire (4); ^c^modified version of the Baby’s Basic Needs Questionnaire (21)

**Supplementary Material**

**Table S2.** Bivariate Pearson correlation coefficients (*r*) for main study variables of interest

|  | Infant negative affect  – age 4 months | Infant regulation  – age 4 months | Infant  FR  – age 4 months | Maternal  FTS  – age 4 months | Maternal  FTS  – age 6 months |
| --- | --- | --- | --- | --- | --- |
| Infant surgency  – age 4 months | 0.21** | 0.56*** | 0.11 | 0.15* | 0.17* |
| Infant negative affect  – age 4 months |  | -0.04 | 0.35*** | 0.28*** | 0.31*** |
| Infant regulation – age 4 months | - |  | -0.15* | -0.002 | -0.07 |
| Infant FR  – age 4 months | - | - |  | 0.39*** | 0.37*** |
| Maternal  FTS  – age 4 months | **-** | - |  | **-** | 0.78*** |

**p* <0.05, ***p* <0.01, ****p* <0.001.

Infant temperament measured via the Infant Behavior Questionnaire-R Very Short Form (31); Food responsiveness (FR) measured via the Baby Eating Behavior Questionnaire (4); Food to soothe measured via the Baby’s Basic Needs Questionnaire (21)

*n*=199-200 for all bivariate associations
